# Ethnobotanical investigation of central and rural villages (neighborhoods) in the Ergani district of Diyarbakır, Turkey

**DOI:** 10.1186/s13002-025-00824-x

**Published:** 2025-11-07

**Authors:** Mustafa Aslan

**Affiliations:** Department of Science Education, Faculty of Education, Osmanbey Campus, Harran Üniversity, Şanlıurfa, 63300 Turkey

**Keywords:** Ethnobotany, Traditional knowledge, Ergani, Upper Mesopotamia, Medicinal plants, Biocultural heritage, Cultural erosion

## Abstract

**Background:**

Ethnobotanical knowledge constitutes a vital component of cultural heritage and biodiversity conservation, particularly in regions undergoing rapid socio-cultural transitions. Southeastern Anatolia, despite its high floristic richness and biocultural diversity, remains underexplored in terms of non-medicinal plant uses.

**Methods:**

This study documents the traditional ethnobotanical knowledge of wild plant species used for non-medicinal and medicinal purposes in the Ergani district of Diyarbakır, Turkey. Data were collected between 2023 and 2024 through structured and semi-structured interviews (*n* = 112), guided fieldwalks, and participant observation across Ergani and 12 surrounding villages. Demographic variables and plant use data were analyzed using descriptive statistics and the cultural importance index to assess the relative significance of each taxon.

**Results:**

A total of 56 plant species belonging to 26 families were identified, used across five categories: medicinal (67.8%), nutritional (53.6%), fuel, decorative, and symbolic (e.g., protection against the evil eye). Multifunctionality was a recurring theme, with several species (e.g., *Vitis vinifera*, *Vitex agnus-castus*) employed in more than two categories. The most represented families were Lamiaceae, Asteraceae, Fabaceae, and Brassicaceae. Wild plants played a central role in local subsistence and cultural practices, with vernacular names and preparation methods varying across gender and age groups. Ritual use, particularly of *Peganum harmala*, remained prevalent among older participants.

**Conclusion:**

The study reveals a complex, integrative system of plant use in Ergani that reflects a holistic ethnoecological worldview. Findings underscore the urgent need for the documentation and preservation of local ecological knowledge amid ongoing socio-economic transformations. Sustaining this intangible heritage is critical for biodiversity conservation, local food security, and cultural resilience.

## Background

Ethnobotany represents a critical interdisciplinary field of research dedicated to investigating the complex relationships between human societies and plants within their cultural and ecological contexts [14]. This discipline systematically examines the diverse ways in which plants are integrated into human life, encompassing not only medicinal applications but also uses for food, shelter, clothing, rituals, and material culture [3]. The convergence of methodologies from botany, anthropology, archaeology, and pharmacology has substantially advanced our comprehension of these intricate relationships while simultaneously underscoring the fundamental importance of traditional botanical knowledge for both cultural heritage preservation and biodiversity conservation [13, 22, 25].

The Anatolian peninsula, recognized as a center of plant diversity, provides an exceptionally rich context for ethnobotanical inquiry. Turkey’s flora comprises approximately 12,000 vascular plant species, with nearly one-third exhibiting endemism, representing one of the most significant biodiversity hotspots in the temperate zone [5, 12]. This remarkable floristic richness is matched by a profound cultural heritage of plant utilization accumulated over millennia of human settlement. However, a systematic analysis of ethnobotanical literature in Turkey reveals a predominant focus on medicinal plants, reflecting broader world wide research trends in pharmacognosy [1, 25]. While this emphasis has yielded valuable pharmacological data, it has inadvertently marginalized the documentation of non-medicinal applications, creating significant gaps in our understanding of holistic plant use systems.

Wild plants in Anatolia fulfill multifaceted roles that extend far beyond therapeutic applications. They serve as crucial resources for nutrition, animal fodder, handicrafts, dyes, and symbolic or ritual practices [8]. These traditional knowledge systems remain particularly vital in rural Anatolian communities, where the seasonal gathering of wild edible shoots, fruits, seeds, and tubers for immediate consumption or preservation through drying, pickling, and other methods continues to represent an important component of local livelihoods and food security [34, 35]. The patterns of plant use demonstrate remarkable spatial heterogeneity, with significant variations observed even across small geographical scales, manifested through distinct vernacular nomenclature, preparation techniques, and application methods [5, 18].

Southeastern Anatolia, characterized by its unique phytogeographical position at the confluence of Iran-Turanian and Eastern Mediterranean floristic regions, supports an exceptional diversity of wild edible and utilitarian plants [7]. The Ergani district of Diyarbakır Province, situated within the historically and ecologically significant region of Upper Mesopotamia, represents an area of particular biocultural importance. This territory constitutes a vital genetic reservoir for wild relatives of key domesticated crops including wheat (*Triticum* spp.), barley (*Hordeum vulgare*), chickpeas (*Cicer* spp.), lentils (*Lens culinaris*), and peas (*Pisum sativum*), highlighting its crucial role in agrobiodiversity conservation and food security [8, 12, 17]. The district hosts culturally diverse populations, primarily Kurdish and Turkish communities, whose distinct ethnobotanical knowledge systems remain inadequately documented [8, 21]. Despite this rich biocultural heritage, comprehensive ethnobotanical research in the Ergani district remains limited, with a pronounced deficiency in the documentation of non-medicinal plant applications [4, 12].

The distinctive contribution of this study within the existing literature previous ethnobotanical investigations conducted in various regions of Turkey provide an essential comparative framework, yet they also delineate the specific gap this study aims to fill. Research in the Espiye district of Giresun Province has documented the diversity of wild plants commercialized in local markets, revealing the economic importance of non-cultivated resources [36]. Parallel studies have focused specifically on ethnoveterinary practices, systematically recording plants used for livestock ailments in the Bingöl and Giresun regions [37, 38]. Furthermore, dedicated research on wild food plants for human consumption has been conducted in provinces including Bingöl and Adana (Karaisalı district) [34, 35].

**Fig. 1:**
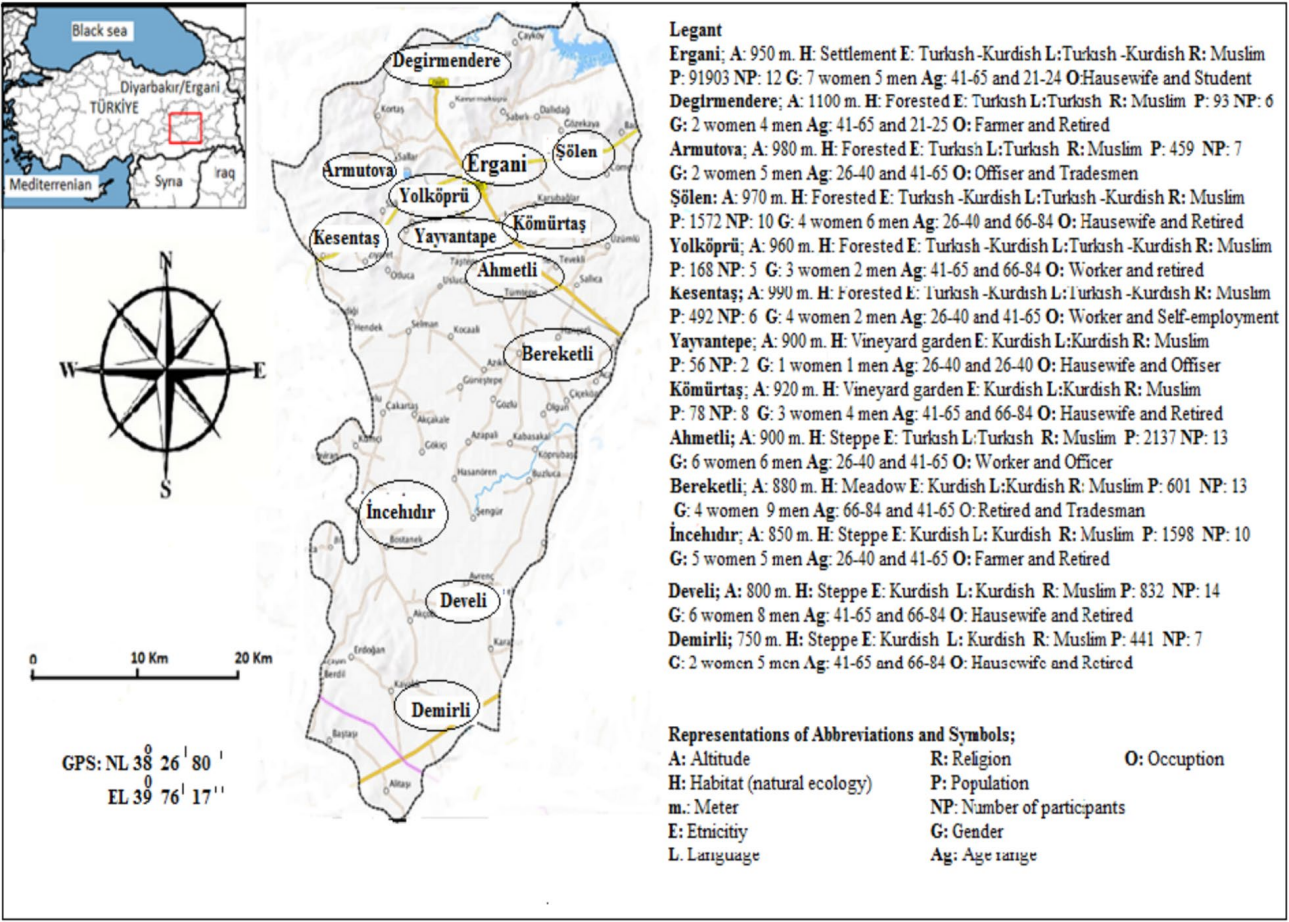
Map of the research area

The primary distinction of the present study lies in its integrated and holistic scope. While the existing literature is often fragmented—focusing on distinct categories such as market plants, ethnoveterinary medicine, or wild foods in isolation—no comprehensive ethnobotanical study in the Ergani district has documented the full spectrum of food, utilitarian, craft, and symbolic plant uses within a unified research framework. This study directly addresses this critical lacuna by systematically investigating both medicinal and, with particular emphasis, non-medicinal applications, which have been historically marginalized. This approach allows for a more complete understanding of the local biocultural system as a whole, rather than its constituent parts. The imperative for this systematic documentation has acquired unprecedented urgency in the context of rapid socio-economic transformations affecting rural Anatolian communities. Urbanization, rural-to-urban migration, agricultural intensification, and the pervasive influences of modernization collectively threaten the intergenerational transmission of this intangible cultural heritage, potentially leading to an irreversible erosion of biocultural diversity [15, 16, 19]. Therefore, this study aims to address these critical knowledge gaps through a systematic ethnobotanical investigation in the Ergani district. The specific objectives are: (1) to comprehensively inventory plant taxa utilized for food, fuel, handicrafts, and symbolic purposes; (2) to document in detail the associated traditional knowledge, including vernacular nomenclature, plant parts used, preparation techniques, and seasonal patterns; (3) to quantitatively assess the cultural significance of documented species using standardized ethnobotanical indices; and (4) to conduct a comparative analysis with previous studies from neighboring districts and broader phytogeographical regions [5, 7, 15, 24, 39] to identify patterns of knowledge distribution and regional variation. This research employs a comprehensive and rigorous methodology to achieve three primary goals: to protect cultural heritage from rapid socio-economic change, to advance the theoretical understanding of biocultural diversity in the Ergani district of Southeastern Anatolia, and to establish an empirical foundation for community-based conservation and sustainable resource management in this historically and ecologically significant region. Detailed site data including name, GPS coordinates, altitude, habitat, ethnicity, language, religion, population, and participant demographics are provided on the accompanying map, with a geographical representation of the study area shown in Fig. 1.

## Methods

### Study design and setting

This ethnobotanical study was conducted from 2023 to 2024 in the Ergani district and 12 surrounding villages in Diyarbakır Province, southeastern Turkey. The rural settlements where the research was conducted are, in order, Şölen, Yolköprü, Demirli, Develi, İncehıdır, Bereketli, Ahmetli, Yayvantepe, Kesentaş, Yolköprü, Armutova, and Degirmendere. The region lies at the intersection of the Mediterranean and Irano-Turanian phytogeographic zones, characterized by semi-arid continental climate and high floristic diversity. The study aimed to document local knowledge on wild and cultivated plant species used for medicinal, nutritional, cultural, and practical purposes.

### Data collection

Data were gathered through structured and semi-structured interviews, participant observation, and guided field walks. Information collected included local plant names, parts used, preparation methods, application modes, and associated cultural beliefs. Socio-demographic data (age, gender, education, occupation) were also recorded. Plant specimens cited by informants were collected, photographed, dried, and identified following standard botanical protocols [6, 12]. Voucher specimens were deposited in the Harran University Herbarium (HARRAN).

### Participants and sampling

A total of 112 local informants (62 men and 50 women), aged 21–84 years, were selected using snowball sampling. Participants were native to the area and identified as knowledgeable about traditional plant use. Prior informed consent was obtained from all participants. Interviews were conducted in Turkish or Kurdish, depending on the respondent’s native language. The research area encompasses a total of 13 settlements, including Ergani as the central locality. The geographical boundaries are defined by GPS coordinates ranging between NL 38° 09′ 18″ and NL 38° 99′ 11″ and EL 39° 77′ 17″ and EL 39° 91′ 11″, with altitudes varying from 750 to 1100 m above sea level. The population structure of the region reflects a balanced distribution of Turkish and Kurdish ethnic groups, with both Turkish and Kurdish widely spoken in nearly equal proportions. The habitats represented in the study area include forested lands, vineyards and gardens, meadows, and steppe ecosystems, indicating ecological diversity. According to demographic data, the total population of the research area is 100,432 inhabitants [33]. From this population, 112 participants were selected for the ethnobotanical survey, consisting of 50 women and 62 men, with ages ranging from 21 to 84 years. During the interview, the name of the place visited in the research area, GPS, altitude, habitat (ecological area), ethnicity, language, religion, population, number of participants, gender, age range, and occupations of participants were given in Table 1.

**Table 1:** Participants’ Location, GPS, Altitude, Habitat (Ecology), Ethnicity, Language, Number of Participants, Gender, Age Range, and Occupation

Location name visited	GPS	Altitude	Ecology	Ethnicity	Language	Population	Number of participants	Gender	Age range	Occupation
Ergani Center	NL 38° 26′ 80″ EL 39° 76′ 17″	950 m	Settlement	Tukısh- Kurdish mixture	Tukısh-Kurdish mixture	91,903	12	7 Woman 5 Man	41–65 21–25	Housewife Student
Degirmendere	NL38° 97′ 21″ EL 39° 77′ 10″	1100 m	Forested	Tukısh	Tukısh	95	6	2 Woman 4 Man	41–65 66–84	Farmers Retired
Armutova	NL38° 97′ 21″ EL39° 82′ 21″	980 m	Forested	Tukısh	Tukısh	459	7	2 Women 5 Man	26–40 41–65	Offiser Tradesmen
Şölen	NL38° 99′ 11″	970 m	Forested	Tukısh- Kurdish mixture	Tukısh- Kurdish mixture	1572	10	4 Women 6 Man	26–40 66–84	Housewife Retired
Yolköprü	NL38° 20′ 12″	960 m	Forested	Tukısh- Kurdish mixture	Tukısh- Kurdish mixture	168	5	3 Woman 2 Man	41–65 26–40	Worker Retired
Kesentaş	NL38° 27′ 70″ EL39° 89′ 13″	990 m	Forested	Kurdish	Kurdish	492	6	4 Women 2 Man	26–40 41–65	Worker Self- employment
Yayvantepe	NL38° 21′ 88″	900 m	Vineyard Garden	Kurdish	Kurdish	56	2	1 Women 1 Man	26–40 26–40	Housewife Officer
Kömürtaş	NL38° 27′ 10″ EL39° 90′ 12″	920 m	Vineyard Garden	Kurdish	Kurdish	78	8	3 Women 5 Man	41–65 66–84	Housewife Retired
Ahmetli	NL38° 27′ 10″ EL 39° 80′ 11″	900 m	Steppe	Tukısh- Kurdish mixture	Tukısh- Kurdish mixture	2137	12	6 Women 6 Man	26–40 41–65	Worker Officer
Bereketli	NL38° 26′ 11″ EL 39° 79′ 16″	880 m	Meadow	Kurdish	Kurdish	601	13	4 Women 9 Man	66–84 41–65	Retired Tradesmen
İncehıdır	NL38° 18′ 13″ EL 39° 89′ 16″	850 m	Steppe	Kurdish	Kurdish	1598	10	5 Women 5 Man	41–65 66–84	Farmers Retired
Develi	NL 38° 26′ 11″ EL 39° 79′ 15″	800 m	Steppe	Kurdish	Kurdish	832	14	6 Women 8 Man	41–65 66–84	Housewife Retired
Demirli	NL 38° 09′ 18″ EL 39° 89′ 12″	750 m	Steppe	Kurdish	Kurdish	441	7	2 Women 5 Man	41–65 66–84	Housewife Retired

### Data classification and analysis

Plants were classified into four use categories: medicinal, food/nutritional, fuel, handicraft, and symbolic (e.g., protection from the evil eye). The cultural ımportance ındex (CI) was calculated for each species to assess its relative cultural significance:

$$\text{CI}=\sum_{i=1}^{i=\text{NU}}\frac{{\text{UR}}_{i}}{N}$$

The index was derived by dividing the usage report (UR) for each designated usage category of a taxon (*i*), spanning from individual usage instances to the total usage number (NU), by the total number of respondents (*N*) in the survey. This quantitative metric provides an indication of the cultural importance of locally recognized species. The theoretical maximum value of this index corresponds to the total number of distinct usage categories [16, 18]. Descriptive statistics were used to analyze demographic data and use-category frequencies. Comparative analysis with regional studies was conducted to contextualize findings. The total number of survey participants was 90. CI_All plants species_ = 10/90 + 32/90 + 23/90 = 0.11 + 0.36 + 0.26 = 0.722.

The contribution index incorporates both the frequency of use, as indicated by the number of informants, and the versatility of each plant taxon, defined by the range of ethnobotanical applications, including medicinal use, nutritional supplementation, charcoal production, ornamental purposes, and protective functions against the evil eye. The theoretical maximum value of the index corresponds to the total number of distinct use categories considered. Plant types are evaluated and ranked based on their prevalence and multifunctionality across these ethnobotanical domains.

**Fig. 2:**
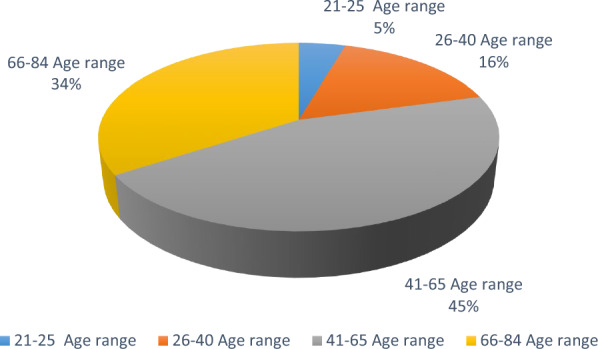
Age distribution of participants who participated in the survey in the research area of Ergani district, Diyarbakır, Türkiye

The age range of the local respondents surveyed in the study area extended from 21 to 84 years. The majority (45%) of participants were within the 41–65 age cohort, followed by 34% in the 66–84 age group, 16% in the 26–40 bracket, and only 5% in the 21–25 range. As illustrated in (Fig. 2), a significant proportion of the sample population (79%) consisted of individuals aged 40 years and above, suggesting that older age groups were more prominently represented in the ethnobotanical knowledge base. The age distribution of local participants in the survey area is shown in Fig. 2. With respect to occupational background, the informants predominantly comprised retirees, agricultural workers, housewives, tradespeople, laborers, civil servants, and self-employed individuals, including those engaged in animal husbandry. This occupational profile is consistent with the region’s socio-economic structure and reflects the interplay between the area’s geographic context, educational attainment, subsistence strategies, and culturally embedded livelihood practices [20, 23].

The ethnobotanical data were categorized into four primary use groups: medicinal, food and nutritional, Firewood, decorative item, and protection from evil eye. The frequency of citation for each plant species was recorded, and plants were organized by family and use category to allow for comparative evaluation with other regional studies. Family, scientific name, local name, part used, ethnobotanical uses, and plant number information regarding plant species in Ergani district are included in Table 2.

**Table 2:** Ethnobotanical uses of the plant species of Ergani district (Symbolic abbreviations *; Herb used in medicine + ; Plants used in food and cooking x, Firewood y; Plant parts are used for decoration and protection against the evil eye. VN. Voucher number)

Family	Scientific name	VN	Local name	Part used	A) Medicinal uses B) Preparation	Ethnobotanical uses
Apiaceae	*Ferula elaeochytris L	MA-864	Çakşır, çakşur Çarpışan	Whole Plant	A) In sexual dysfunction B) Decoction	The whole plant is harvested, mainly for pickling. Local people believe that İts pickled form enhances sexual function in men Harvesting usually occurs in spring, and it is fermented in vinegar or salty water without drying
Apiaceae	*Eryngium campestre L	MA-865	Kerbeş, Şekıran, Eşek dikeni	Above ground	A) Muscle relaxant disorders B) Decoction	The plant’s above-ground parts are used to relax muscles and ease fatigue. People usually make a decoction from fresh or lightly dried parts and massage it onto their muscles
Apiaceae	*Ammi visnaga (L.) Lam	MA-866	Dıdana giya Kürdan	Seeds, flowers	A) Muscle relaxant disorders B) Infusion	Used to relax muscles and reduce tiredness. People usually make a tea from the seeds and flowers, which can be drunk or rubbed onto muscles
Asteraceae	*Anthemis arvensis L	MA-867	Papatya, Derman	Flower	A) Muscle relaxant disorders B) Infusion	The plant is commonly used to relax muscles or ease mild aches. People usually make a tea or infusion from the flowers and leaves, or apply it byrubbing onto the skin
Asteraceae	*Achillea oligocephala DC	MA-868	Civanperçemi, Kurpotu, Sporiş	Above ground	A) Muscle relaxant disorders B)Infusion	Used to ease muscle aches and provide relaxation. People can make tea from the flowers and leaves or apply it by massaging onto the muscles
Asteraceae	*Bellis perennis L	MA-869	Koyungözü, Çavemi	Flowers and leaves	A)Stomach diseases, wounds B)Infusion, externally	Commonly used to soothe the stomach and help heal wounds. People make a tea from the flowers or crush them and apply directly onto the skin
Asteraceae	*^,+^*Carthamus tinctorius* L	MA-870	Haspir, Aspire Zerdeotu	Flowers	A) Blood circulation failure B) Infusion	The flowers are often used as a natural dye and to add color and flavor to food People also drink them as a tea to improve blood circulation and ease pain
Asteraceae	+ *Gundelia tournefortii* L	MA-872	Kenger, Kereng	Young Shoots, leaves, and root gum	Not for medical use	The young shoots are eaten as a vegetable, either cooked or raw. The root produces a special gum called “kenger gum,” which people chew and be is good for dental health. It is also thought to stimulate appetite and aid dig believe digestion
Asteraceae	^+^*Tragopogon longirostris* L	MA- 873	Şıng, Pısç Yemlik	Young shoots, leaves	Not for medical use	The young shoots and leaves are collected in spring and eaten fresh or cooked in meals. Local people especially value it in spring as a nourishing and strengthening food
Amaranthaceae	^+^*Amaranthus retroflexus* L	MA-874	Levendur, Somaz	Shoots and leaves	Not for medical use	The leaves and shoots are cooked or sautéed as a vegetable People consider it a nourishing food, especially popular in summer meals
Anacardiaceae	+ *Rhus coriaria* L	MA-871	Sumak, Sımok	Fruits (drupes)	Not for medical use	The ripe fruits are dried and used as a spice in cooking. In some areas, they are also used to make vinegar or sauces, adding a tangy flavor to dishes
Anacardiaceae	*^,+^*Pistacia terebinthus* L	MA-875	Menengiç, Çitlenbik	Fruits and resin	A) Healing, wounds B) Crushed resin is applied	The fruits are usually roasted and eaten as snacks or used to make coffee The resin is applied to heal wounds or used for massage
Brassicaceae	^+^*Eruca sativa* L	MA-876	Roka, Acice, Şelmok	Leaves and Young shoots	Not for medical use	The leaves and shoots are used in salads or cooked dishes. Locally, it is known to aid digestion and stimulate appetite
Brassicaceae	*^+^*Lepidium sativum* L	MA-877	Dejnık, Tere	Leaves and	A) In digestion and Immune B)Seed is crushed with honey	The leaves and seeds are eaten raw or in salads. It is also known to aid digestion and support the immune system
Brassicaceae	^+^*Nasturtium officinale* L	MA-878	Tuzık, Suteresi	Leaves and Shoots	Not for medical use	The leaves and shoots are eaten raw in salads or cooked dishes. Locally, it is known to be refreshing, aid digestion, and stimulate appetite
Brassicaceae	*Capsella bursa-pastoris (L.) Medik	MA- 885	Çobançantası Nançuçık	Leaves and Young shoots	A)Stop the bleeding B) Decoction	The leaves and shoots are eaten raw or cooked in salads and dishes. Locally, it is known to help stop bleeding and aid digestion
Brassicaceae	^+^*Sinapis arvensis* L	MA-879	Ğerdel, Hardal	Leaves and Seeds	Not for medical use	The leaves are eaten in salads or cooked dishes, and the seeds are used as a spice. Locally, it is known to aid digestion and stimulate appetite
Cyperaceae	*Cyperus rotundus L	MA-880	Topalak, Hasırotu	Roots and	A)Digestive and gas remuval B) Infusion	The roots and rhizomes are made into a tea or decoction to help with digestive issues and provide a soothing effect. It is known to relieve gas and promote relaxation
Equısetaceae	*Equisetum arvense L	MA-881	Atkuyruğu, Kırkkilitliot	Aerial stems	A) Urinary tract disease B) Infusion	The stems are made into a tea or infusion to strengthen bones and support urinary health
Fabaeceae	^+^*Cicer echinospermum* L	MA-882	Nok, Nohut Leblebi	Seeds	Not for medical use	The seeds are usually cooked or ground and added to dishes. Locally, they are known to be nutritious and energizing
Fabaeceae	*Glycyrrhiza glabra L	MA-833	Ava suse, Meyan, Biyam	Roots	A) Cold and Cough B) Decoction	The roots are chewed for their sweet flavor or prepared as a tea. Locally they are used to soothe coughs and sore throats, aid digestion, and provide a refreshing effect
Fabaeceae	^+^*Lathrus sativus* L	MA-884	Şollık, Çolban Mürdümük	Seeds	Not for medical use	The seeds are usually cooked in meals, added to soups or mixed with bulgur Locally, it is known as a filling and nutritious legume
Fabaeceae	^+^*Pisum sativum* L	MA-885	Bekle, Bezelye	Seeds (peas)	Not for medical use	The seeds are used as a vegetable in meals, added to soups or dishes. Locally, they are known to be nutritious and energizing
Fabaeceae	^+^*Lens culinaris* L	MA-886	Nisk, Mercimek	Seeds (lentils)	Not for medical use	The seeds are used in meals, especially in soups and dishes. Locally, they are known to be nutritious and energizing
Fabaeceae	*^+^*Trigonella foenum-graecum* L	MA-887	Tamğeş	Seeds	A) Diabetes disease B) İnfusion	The seeds are used as a spice in cooking or prepared as a tea. Locally, they are known to stimulate appetite, aid digestion, and help regulate blood sugar
Boragınaceae	^+^*Anchuza azurea* L	MA-888	Guruz	Leaves and	Not for medical use	The leaves and flowers are usually brewed as a tea or used in cooking. Locally, they are known to aid digestion and provide a calming effect
Fagaceae	*^,x^ *Quercus brantii* L	MA-889	Çılo, Mazi, Palamut	Fruits (acorns)	A) Diarrhea and digestive B) İnfusion	The acorns are roasted or ground and used in meals or mixed with flour. sometimes brewed as a tea or decoction to help with diarrhea and digestive issues. It used as firewood
Hyperıcaceae	*Hypericum perforatum L	MA-834	Kantaron, Kantül	Flowers and leaves	A) Psychiatric treatment and wounds B) İnfusion and externally	The flowers and leaves are usually brewed as a tea or made into an oil for topical use. Locally, it is known to improve mood, provide relaxation, and help heal wounds
Lamıaceae	*Lavandula stoechas L	MA-833	Serreş, Lavanta	Flowers	A) Stress and İnsomnia B) Decoction	The flowers are dried and used as tea or in sachets for their fragrance Locally, they are known to reduce stress and help improve sleep
Lamıaceae	*^,+^*Mentha longifolia* L	MA-891	Nane, Rıho	Leaves	A) Indigestion B) İnfusion	The leaves are brewed as tea or used to flavor dishes. Locally they are known to aid digestion and provide a refreshing effect
Lamıaceae	*^,+^*Mentha piperita* L	MA-892	Rıhan, Reyhan	Leaves	A) Indigestion B) İnfusion	The leaves are brewed as tea or used to flavor dishes. Locally, they are known to aid digestion, refresh, and freshen the breath
Lamıaceae	*^,+^*Mentha pulegium* L	MA- 893	Pıng, Pung Yarpuz	Leaves	A) Indigestion B) İnfusion	The leaves are brewed as tea or used to flavor dishes. Locally, they are known to aid digestion, refresh, and provide a calming effect
Lamıaceae	^+,y^ *Ocimum basilicum* L	MA-894	Rıyhan, Rıho Reyhan	Leaves and stems	Not for medical use	The leaves are used as a spice in cooking or brewed as tea. It is also valued for its pleasant fragrance and decorative appearance in homes and ornamental plant
Lamıaceae	*^,+^*Rosmarinus officinalis* L	MA-895	Biberiye Kuşdili	Leaves and stems	A) Indigestion and Vitality B) İnfusion	The leaves are used as a spice in cooking or brewed as tea. Locally, it is known to aid digestion and improve memory and mental alertness It is also valued for its pleasant fragrance and decorative appearance in homes
Lamıaceae	*^,+^*Thymbra spicata* L	MA-896	Zahter Kekik	Leaves and flowers	A) Indigestion and B) İnfusion	The leaves and flowers are brewed as tea or added to dishes as a spice. Locally, it is known to aid digestion, refresh, and freshen the breath
Lınaceae	*Linum usitatissimum L	MA-897	Keten, Beziryağı	Seeds	A) Indigestion B) the seed is eaten raw	The seeds are used in cooking or brewed as tea, and flaxseed oil is also extracted. Locally, it is known to aid digestion and provide nutrition
Liliaceae	^+^*Asphodelus aestivus* Brot	MA-898	Gullık, Çiriş Kişkişotu	Roots and tubers	Not for medical use	The roots and tubers are usually cooked or boiled and eaten. Locally, they a are known to be nutritious and energizing
Liliaceae	^+^*Ornithogalium narbonense* L	MA-899	Ğazring, Akbaldır	Roots and tubers	Not for medical use	The roots and tubers are usually boiled or cooked in meals. Locally, they are known to be nutritious and energizing
Liliaceae	*^,+^*Allium nigrum* L	MA-815	Sirım, Sarımsak Kara soğan	Roots and tubers	A) Cold and cough B) the tuber crushed	The bulbs and leaves are used as a spice or vegetable in cooking. Locally it is also as a natural remedy to boost immunity and help relieve colds coughs, and other minor ailments
Malvaceae	*^,x^*Alcea hohenackeri* Boiss	MA-816	Kuşburnu, Hiro	Flowers, roots, leaves, steems brances	A) Cold and cough in wounds B) İnfusion, mash	The flowers are boiled to prepare syrup or tea, traditionally used against cough, and sore throat, especially for children’s night cough. Leaves are applied as a poultice on wounds and swellings, while in some villages the plant is also cultivated ornamentally, its stem is used firewood and as
Malvaceae	*Malva sylvestris L	MA-818	Ebegümeci, Kömeç, Gomeç	Leaves, Flowers and roots	A) Sore throat, expectorant inflament boil B) İnfusion, mash	In spring, fresh leaves are collected and used as a filling in pastries and flatbreads It is also prepared as a soup. The dried flowers are brewed into tea, consumed It is also prepared as a soup. The dried flowers are brewed into tea, consumed as a throat soother and expectorant. A poultice made from the roots is applied on boils and skin wounds
Nıtrarıaceae	*^y^*Peganum harmala* L	MA-819	Nazarlık Özerlik Şeytankaçıran	Seeds	A) Gynecological diseases, indigestion, insomnia B) İnfusion, mash	Harmala seeds are burned as incense, especially around newborns, to ward off evil and negative energies. Boiled ones are used for fertility and women's health, while heated ones are beneficial for rheumatism and joint pain. The seeds are pierced through the center, threaded and strung together to create decorative ornaments They are hung in homes to ward off the evil eye
Polygonaceae	^+^*Rumex acetosella* L	MA-814	Kuzukulağı Tırşo	Leaves	Not for medical use	Fresh leaves are eaten as a vegetable in salads or cooked dishes. Leaves are also boiled as tea for digestive support and stomach relief. Traditionally, it is used asan appetite stimulant
Polygonaceae	^+^*Rheum ribes* L	MA-821	Işgın, Uçkun	Young stem	Not for medical use	Young shoots are collected in spring, peeled, and eaten raw or cooked in traditionally used to aid digestion and regulate bowel movements
Portulacaceae	*^,+^*Portulaca oleraceae* L	MA- 822	Pirpirim, Semizotu, Pırpar	Parts of the plant	A) Constipation B) Decoction	Leaves and young shoots are collected and consumed fresh in salads or cooked dishes. They are valued for their slightly sour taste and nutritional properties. Traditionally, they are also used to support digestion and as a mild laxative
Pinaceae	+ ^,x^*Pinus pinea* L	MA-823	Çam fıstığı Fıstığa çem	Seeds, trunk branches	Not for medical use	The seeds (pine nuts) are collected and consumed raw or roasted. They are used in cooking and traditional recipes for their flavor and nutritional value. The branches and trunk are used as firewood
Ranunculaceae	*Nigella sativa L	MA- 824	Çörek otu, Tohuma reş, Siyah kimyon	Seeds	A) Gynecological diseases B) the seeds are crushed	Seeds are used as a spice in bread, pastries, and desserts, and given to children breastfeeding mothers to increase milk. Regular consumption may reliev menstrual pain, act as a diuretic, strengthen immunity menstrual
Rosaceae	*Alchemilla vulgaris L	MA-825	Aslanpençesi, Hanımpelerini	Flowers and Leaves	A) Gynecological diseases and menstrual irregular B) İnfusion	Leaves are collected and prepared as tea to relieve menstrual pain, regulate menstruation, and support women’s reproductive health. Traditionally, it is also used to aid digestion and as a mild diuretic
Rosaceae	*^,+^*Cerasus mahaleb* L	MA-826	Mahlep, Enderiz, Keniro	Fruits and seeds	A) Diabetes and Indigestion B) İnfusion	Fruits are dried and used to flavor pastries and desserts. Seeds are added to dough for texture and taste. Traditionally, consumed as tea or paste to regulate blood sugar and support digestion
Rosaceae	*^,+,x^*Crataegus aronia* L	MA- 827	Guviç, Alıç Sızar	Fruit and leaves	A) Cholesterol and heart diseases B) İnfusion	Fruits and dried leaves are prepared as tea or syrup to reduce cholesterol and blood pressure. Branches and trunk are used as firewood. Traditionally fruits and tea are also consumed to support heart health
Rosaceae	*Rosa canina L	MA-828	Kuşburnu, Gul, Şilan	Flowers, fruits,	A) Cold flu and İmmunity B) İnfusion	Fruits and flowers are dried and brewed as tea for flu, colds, and immunity support are also made into marmalade and jam; flowers are used decoratively
Ulmaceae	*^,y^*Celtis tournefortii* L	MA- 829	Dardağan, Dığdıgo, Çıtlenbik	Fruits	A)Diabetes and blood pressure lowering B) the fruits are crushed	Ripe fruits are eaten fresh or dried and stored for winter; used as tea or eaten to regulate blood sugar and blood pressure. Branches, storage accessories, and craft pieces are used as firewood or tied and hung around children and homes to ward off
Urtıcaceae	*Urtica dioica L	MA-830	Gezgezog Isırganotu	Flowers Leaves, fruits	A) Indigerter and Urinary B) Decoction	Leaves and fruits are used to treat various ailments, including digestive issues, urinary problems, and as a general tonic. Traditionally, it is also consumed to support blood purification and overall health
Verbenaceae	*^,x^*Vitex agnus-castus* L	MA- 813	Hayıt, İffetağacı	Fruits, s	A) Gynecological diseases and menstrual irregular B) Decoction	The stems of Vitex agnus-castus are primarily used as firewood. In addition the plant holds traditional medicinal value; various parts, including fruits and, leaves, have been used to support women’s reproductive health, regulate menstrual cycles and relieve premenstrual symptoms
Vıtaceae	*^,+,x^*Vitis vinifera* L	MA-832	Tıri, asma Üzüm	Soots, leaves, Fruit, seed, branch and trunk	A)-Diabetes and Indigestion B) the seeds are crushed, İnfusion	The shoots are consumed as vegetables, and leaves are used especially for wrapping (sarma). The fruits are eaten fresh, dried for winter, or processed into fruit leather, molasses, and syrup. The seeds are ground and consumed as tea or flour, traditionally believed to regulate blood sugar and aid believed to regulate blood sugar and aid digestion. The trunk and branches are used as firewood. Grape molasses is consumed as an immunity booster and to relieve colds

## Results

A total of 56 plant species were documented as being used ethnobotanically by local communities in the Ergani district. These species were categorized based on their reported uses, which included medicinal, nutritional/culinary, fuel (firewood), decorative, and symbolic purposes such as protection against the evil eye. Medicinal use was the most commonly reported category, with 38 plant species (67.8%) cited for treating various ailments. Among these, 20 species were used exclusively for medicinal purposes, while were reported to have both medicinal and nutritional/culinary applications. Additionally, 4 species served both medicinal and firewood purposes, and 2 species were used across three functions: medicinal, nutritional/culinary, and as firewood. Another 2 species were cited for combined medicinal, firewood, and dietary use, reflecting the multifunctional value attributed to certain taxa.

**Fig. 3:**
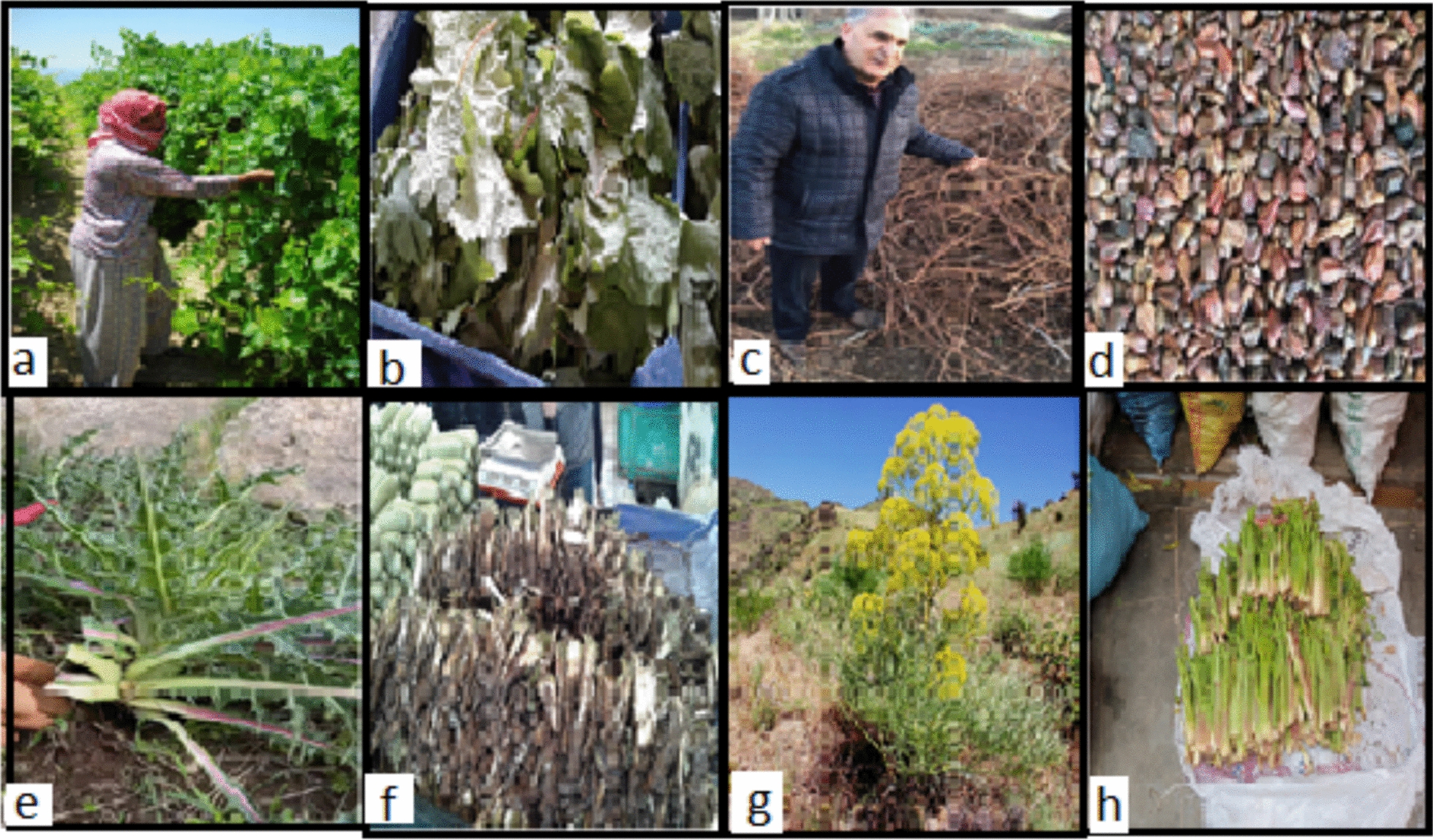
**a** Forager harvesting asma leaves (*Vitis vinifera*) in a vineyard. **b** A leaves being sold at a market. **c** Asma stems and branches stored as firewood by the researcher. **d** Asma seeds available for purchase at an herbalist’s shop. **e** The Kenger plant (*Gundelia tournefortii*) being gathered in the wild. **f** Fresh Kenger shoots offered for sale in the market.** g** The Çakşır plant (*Ferula elaeochytris*) collected from its natural habitat. **h** young Çakşır shoots sold fresh at the market

Nutritional and culinary uses were associated with 30 species (53.6%), with 14 species used solely for food and dietary supplements. This category also included species with overlapping functions: 12 species with medicinal uses, and several others also employed for firewood or decorative purposes, illustrating a complex overlap between food and health practices. The most significant plant species documented in the study area, including those harvested from the wild and sold in local markets, are presented with photographs in Fig. 3.

**Fig. 4:**
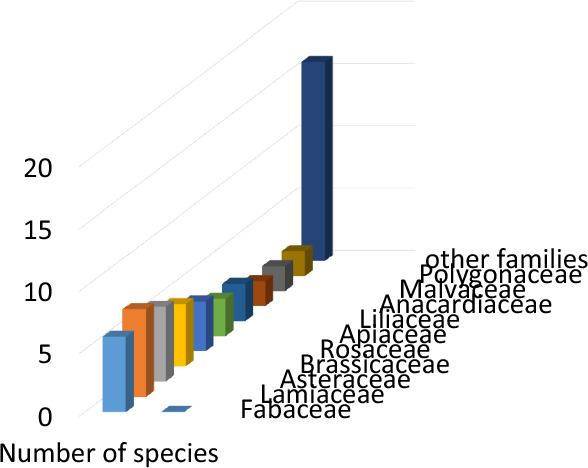
Distribution of plant species defined as ethnobotanical in the research area according to their families

Decorative and symbolic uses were less frequent but culturally significant. One species was reported to be used exclusively as an amulet and decorative item, while another was used for nutrition, decoration, and protection against the evil eye. One species was used for decoration and spiritual protection, and another for firewood and symbolic protection. These findings highlight the integration of symbolic beliefs into daily plant use, particularly in protective and ritual contexts. The multifunctionality of plant species is a notable aspect of ethnobotanical knowledge in Ergani. Several species were employed in more than two use categories, indicating that local ethnobotanical practices are not strictly compartmentalized but rather reflect a holistic understanding of plants as therapeutic, nutritional, utilitarian, and symbolic resources. It was determined that the leaves, fresh shoots, and/or stems of numerous wild plant species are consumed as food in the Ergani region, species such as *Gundelia tournefortii*, *Eruca sativa*, *Lepidium sativum*, *Nasturtium officinale*, *Rumex acetosella*, *Ornithogalium narbonense, Allium nigra, Sinapis arvensis*, *Cicer echinospermum*, *Lathyrus sativus*, *Pisum sativum*, *Anchusa azurea*, *Mentha longifolia*, *Tragopogon longirostris, Portulaca oleracea*, *Vitis vinifera*, *Rumex acetosella*, *Asphodelus aestivus Amaranthus retroflexus,* and *Malva sylvestris* are traditionally consumed in a variety of local dishes. These plants play a significant role in the region’s culinary heritage, reflecting both their nutritional value and the local population’s deep-rooted knowledge of wild edible flora. Figure 3 illustrates the collection and commercialization of wild edible and medicinal plants by local inhabitants, who gather these resources from their natural habitats and sell them in regional markets. Medicinal plants represent the most extensively utilized category of plants among the local population. Their widespread use reflects the deep-rooted traditional knowledge and cultural reliance on natural remedies for the treatment and prevention of various ailments. Notable examples of medicinal plant species recorded in the study include *Ferula orientalis*, *Eryngium campestre*, *Ammi visnaga*, *Anthemis arvensis*, *Achillea oligocephala*, *Bellis perennis*, *Helianthus annuus*, *Capsella bursa-pastoris*, *Cyperus rotundus*, *Equisetum arvense*, *Glycyrrhiza glabra*, *Quercus brantii*, *Hypericum perforatum*, *Lavandula stoechas*, *Rosmarinus officinalis*, *Linum usitatissimum*, *Plantago lanceolata*, *Pinus pinea*, *Alchemilla vulgaris*, *Cerasus mahaleb*, *Crataegus aronia*, *Rosa canina*, *Celtis tournefortii*, *Urtica dioica*, *Vitex agnus-castus*, *Vitis vinifera*, *Mentha pulegium*, and *Mentha piperita*. Several plant species have been traditionally utilized as firewood by local communities in the study area. Notable among these are *Quercus brantii*, *Vitex agnus-castus, Althea hohenackeri, Vitis vinifera, Crataegus aronia*, and *Pinus pinea*. The selection of these species is primarily based on their availability, wood density, and burning efficiency, underscoring their significance in fulfilling domestic energy requirements, particularly in rural and semi-rural settings. An important finding of the study is the use of plant parts from *Peganum harmala*, *Ocimum basilicum*, and *Celtis tournefortii* in the production of decorative items, which are traditionally believed to provide protection against evil spirits and the evil eye. Furthermore, the burning of *Peganum harmala* as incense is commonly regarded as a ritual practice to ward off the evil eye. Commonly cited species included *Foeniculum vulgare*, *Gundelia tournefortii*, *Peganum harmala*, and *Achillea oligocephala*. Local knowledge exhibited significant intergenerational and gender-based variation. Several plants were reported to have multipurpose uses, and many were gathered from the wild rather than cultivated. Ritual and protective plant use (e.g., against the evil eye) remains prevalent in rural areas. These plants hold symbolic and cultural significance, reflecting the intertwining of aesthetic use and folkloric beliefs in local ethnobotanical practices. The distribution of plant species by family within the study area is illustrated in Fig. 4.

A total of 56 plant species, classified under 26 distinct botanical families, were documented in the ethnobotanical study conducted in the research area. The most commonly represented families include Lamiaceae (7 species, 12.5%), Asteraceae and Fabaceae (6 species, 10.7% each), Brassicaceae (5 species, 8.9%), Rosaceae (4 species, 7.1%), Liliaceae and Apiaceae (3 species, 5.1% each), and Anacardiaceae, Malvaceae, and Polygonaceae (2 species, 3.5% each). The remaining families are represented by a single species each. This distribution indicates that these dominant plant families hold a significant place in the local community’s traditional ethnobotanical knowledge and practices. The ethnobotanical distribution of plant taxa by family in the study area aligns closely with findings from nearby regions. For instance, comparable results were reported in the neighboring district of Çermik. The ethnobotanical use of plants in the Ergani and Çermik districts shows a similar percentage distribution. In the Çermik district, the most commonly used plant families include Lamiaceae (8 species, 13.1%), Asteraceae (6 species, 11.4%), Fabaceae (7 species, 12.1%), Brassicaceae (5 species, 10.1%), Rosaceae (4 species, 8.7%), and Liliaceae (3 species, 6.5%). Additionally, Anacardiaceae, Malvaceae, and Polygonaceae are each represented by 2 species (5.1%) [5]. The ethnobotanical survey in the Ergani district demonstrates a diverse knowledge system in which plants are employed for functional and symbolic purposes. Medicinal uses were dominant, with nearly two-thirds of species cited for therapeutic purposes, reflecting wider Anatolian and Eastern Mediterranean reliance on natural remedies where cultural continuity and limited healthcare access persist. A notable feature is the multifunctionality of plants, many serving simultaneously medicinal, nutritional, and utilitarian roles. Species such as Vitis vinifera and *Vitex agnus-castus* exemplify this overlap, indicating ecological pragmatism and adaptive knowledge responsive to resource availability and seasonal cycles. Nutritional use was also prominent, with 30 wild species incorporated into local diets. Preferences for leaves, shoots, and stems reflect culinary traditions and nutritional considerations. Decorative and symbolic applications, though less common, underscore cultural values: *Peganum harmala*, *Ocimum basilicum*, and *Celtis tournefortii* are employed for spiritual protection, linking botanical knowledge to ritual and metaphysical domains. Firewood use of taxa such as *Quercus brantii* and *Pinus pinea* demonstrates deliberate species selection based on fuel quality and availability, highlighting the role of plants in subsistence practices. Variation in knowledge across generations and genders indicates uneven distribution shaped by social roles and experience. The predominance of wild plant gathering further underlines the interdependence of cultural knowledge and natural landscapes, reinforcing the importance of conserving both biodiversity and traditional practices.

Comparative analysis with previously published ethnobotanical studies across Turkey reveals both thematic continuities and significant regional particularities. Like prior research conducted in Çermik [5], Van [26], Suruç [24], Maden [2], Balkans (Eastern Europe) [27], and Central Anatolia [9], the Ergani study confirms that wild plant species are primarily used for medicinal and nutritional purposes. Widely cited species such as *Urtica dioica*, *Mentha longifolia*, *Malva sylvestris*, *Rhus coriaria*, *Glycyrrhiza glabra, Alcea hohenackeri*, *Alchemilla vulgaris*, *Vitis vinifera*, *Portulaca oleraceae*, *Rheum ribes,* and *Gundelia tournefortii* appear consistently across these regions, suggesting the existence of a core ethnobotanical repertoire that transcends provincial boundaries. However, the current findings also highlight local specificity. The ritualistic and symbolic use of species like *Peganum harmala*, burned as incense for spiritual protection, and *Celtis tournefortii*, used in the preparation of amulets, reflect a more pronounced integration of plant use into cosmological and apotropaic traditions in Ergani than typically reported in adjacent regions. Furthermore, multifunctional taxa such as *Vitex agnus-castus*, employed for medicinal, fuel, and symbolic purposes, underscore a distinctly utilitarian ethnoecological logic that aligns closely with similar findings from Kurdish-inhabited areas in Iraq [28]. While medicinal uses have historically dominated ethnobotanical literature in Turkey [29], recent studies especially those aligned with biocultural and eco-cultural heritage frameworks have emphasized the importance of recognizing multifunctionality in plant usage [30]. In this context, the present study contributes novel insights by documenting the commercial role of wild edibles in rural markets, the persistence of plant-based spiritual practices, and the continued reliance on plant biomass for domestic energy elements often underrepresented in classical ethnopharmacological studies. Ergani’s diverse applications of plant taxa thus reflect both ecological pragmatism and cultural embeddedness. Compared with Mediterranean Turkey, where culinary traditions often center on cultivated herbs and semi-wild greens [31], Ergani’s stronger reliance on uncultivated flora reflects an adaptive subsistence strategy conditioned by historical marginalization, arid geography, and limited access to formal healthcare and infrastructure. From a cultural standpoint, the findings reinforce the argument that ethnobotanical knowledge in Southeastern Anatolia is not merely utilitarian, but deeply woven into the social fabric, symbolic systems, and intergenerational knowledge transmission practices. The prominence of spiritual and protective plant uses particularly among older women parallels similar patterns observed in the Balkans [27]. Such uses illustrate how plants act as mediators between the material and metaphysical realms, carrying meanings that go beyond pharmacology or nutrition. Additionally, the gendered and age-based stratification of knowledge, where elder community members act as custodians of ecological memory, echoes cross-cultural patterns reported in indigenous and rural societies globally [30, 32]. As such, this study highlights not only the ecological role of plants but also their embeddedness in cultural resilience, local identity, and symbolic continuity under conditions of socio-economic transition and globalization.

## Discussion

The ethnobotanical findings from the Ergani district reveal a complex and dynamic knowledge system where plants are deeply embedded in the cultural and ecological fabric of daily life. This study not only inventories useful taxa but also illuminates a holistic ethnoecological worldview where the boundaries between medicinal, nutritional, and symbolic uses are fluid and context-dependent. Our discussion situates these findings within the broader frameworks of biocultural diversity, knowledge transmission, and the pressing challenges of socio-ecological change in Southeastern Anatolia.

The predominance of medicinal applications, representing 67.8% of the documented species, aligns with global ethnobotanical trends where therapeutic knowledge is often the most salient and well-preserved facet of traditional ecological knowledge (TEK) [1, 2]. This reliance underscores the role of local phytotherapy as a primary or complementary healthcare strategy, particularly in rural settings where access to formal biomedical services may be limited or economically burdensome [3]. The prevalence of plants addressing digestive (e.g., *Mentha* spp., *Thymbra spicata*), respiratory (e.g., *Glycyrrhiza glabra*, *Malva sylvestris*), and gynecological issues (e.g., *Vitex agnus-castus*, *Alchemilla vulgaris*) reflects common health concerns managed within the domestic sphere. Notably, the use of species such as *Peganum harmala* for spiritual and gynecological purposes and *Ferula elaeochytris* as a male sexual tonic points to a pharmacopoeia that addresses both physiological and socio-somatic aspects of health, consistent with findings from other Kurdish-inhabited areas and the wider Upper Mesopotamia [4, 5].

A central finding of this research is the pronounced multifunctionality of many plant species, which reflects a sophisticated, integrated knowledge system that maximizes utility within ecological and economic constraints [9]. For instance, *Vitis vinifera* serves as a quintessential example: its shoots and leaves are consumed as food, its fruits are processed into preserves and molasses, its seeds are used medicinally, and its pruned branches provide fuel. Similarly, *Quercus brantii* provides acorns for food and medicine, while its dense wood is a valued fuel source. This multifunctionality represents a key adaptive strategy that enhances community resilience; by relying on a single species for multiple needs, communities can buffer against seasonal shortages and ecological perturbations [10].

The documentation of 30 wild food species (53.6%) highlights their critical role in ensuring food security and dietary diversity. The practice of collecting wild greens, known locally as “sıgın” (e.g., *Rumex acetosella*, *Gundelia tournefortii*, *Eruca sativa*), is a vibrant culinary tradition that provides essential micronutrients and reinforces a connection to seasonal cycles and local landscapes [11, 34]. The commercialization of these edibles in local markets points to their socio-economic importance, yet also raises sustainability concerns. Overharvesting of popular species like *Gundelia tournefortii* could lead to local population declines, as observed elsewhere in Turkey [12, 39], necessitating the development of sustainable harvesting guidelines and community-based management.

The persistence of symbolic uses, particularly protection against the evil eye (“nazar”), demonstrates the deep cultural embedding of flora. *Peganum harmala* is again prominent for its dual role as a medicinal and powerful apotropaic agent, with practices like seed-burning for purification and amulet-making possessing ancient roots in Upper Mesopotamia [4, 13]. These rituals are active components of a cultural system that uses botanical elements to manage psychological stress and foster social cohesion [14]. Their reported prevalence among older informants signals a vulnerability, as such symbolic knowledge is often the first to erode with modernization, representing a significant loss of intangible cultural heritage.

Our findings confirm a critical threat to this biocultural heritage: an intergenerational knowledge gap, with detailed expertise concentrated among older generations [15]. This erosion is driven by rural-to-urban migration, the transition to cash-based economies, the availability of modern commodities, and shifts in educational priorities. Furthermore, we observed a gendered dimension to knowledge; women typically held deeper expertise in medicinal plants and wild greens for daily sustenance, while men were more knowledgeable about species used for fuel, construction, and animal husbandry [16, 32]. This underscores the necessity of inclusive ethnobotanical research to obtain a comprehensive picture.

The ethnobotanical data carry urgent implications for both environmental sustainability and public health policy. The documented reliance on wild taxa indicates a knowledge system finely attuned to local landscapes, yet one that is increasingly threatened by anthropogenic pressures such as overgrazing, land conversion, and climate change. Multifunctional species like *Gundelia tournefortii*, *Glycyrrhiza glabra*, and *Hypericum perforatum* are highly sensitive to habitat fragmentation, meaning that ecosystem degradation directly disrupts local health and food security pathways. Therefore, integrating TEK into conservation planning through participatory mapping, community-based resource management, or ethnobotanical buffer zones is essential for enhancing socio-ecological resilience.

Ultimately, the preservation of ethnobotanical heritage in Ergani is not merely a cultural priority but a socio-ecological necessity. Future efforts must be multifaceted, focusing on: (1) in-situ conservation and sustainable harvesting protocols, (2) ex-situ conservation in home gardens, (3) knowledge revitalization through educational programs and digital archives, and (4) phytochemical and pharmacological validation of key species to scientifically appraise traditional uses and potentially contribute to new therapeutic discoveries.

## Conclusion

This study highlights the rich ethnobotanical knowledge held by local communities in the Ergani district, where plant species are used in diverse and overlapping ways for medicinal, nutritional, utilitarian, and symbolic purposes. Medicinal use emerged as the most dominant category, reflecting a deep-rooted reliance on traditional healthcare practices. However, the multifunctionality of many species serving as food, fuel, medicine, and cultural symbols underscores the integrated nature of local ecological knowledge. The findings also reveal that wild plants continue to play a vital role in food security, cultural identity, and domestic economies, particularly through foraging and market-based trade. Symbolic uses, such as protection against the evil eye, remain culturally significant, demonstrating that spiritual beliefs continue to shape plant use practices alongside subsistence needs. This ethnobotanical versatility reflects a holistic worldview, wherein health, sustenance, ritual, and environment are interconnected. Preserving such knowledge is essential not only for biodiversity conservation but also for sustaining the cultural heritage and resilience of rural communities. Continued documentation and recognition of local plant knowledge will contribute to its transmission across.

## Data Availability

No datasets were generated or analysed during the current study.
